# Impact of anti-thymocyte globulin dose for graft-versus-host disease prophylaxis in allogeneic hematopoietic cell transplantation from matched unrelated donors: a multicenter experience

**DOI:** 10.1007/s00277-021-04521-z

**Published:** 2021-05-04

**Authors:** Sara Butera, Marco Cerrano, Lucia Brunello, Chiara Maria Dellacasa, Danilo Giuseppe Faraci, Sara Vassallo, Nicola Mordini, Roberto Sorasio, Francesco Zallio, Alessandro Busca, Benedetto Bruno, Luisa Giaccone

**Affiliations:** 1 Department of Oncology, SSD Trapianto Allogenico di Cellule Staminali, A.O.U. Città della Salute e della Scienza di Torino, Via Genova 3, 10126 Torino, Italy; 2 Department of Molecular Biotechnology and Health Sciences, Division of Hematology, University of Torino, Torino, Italy; 3 Department of Oncology, Division of Hematology, A.O.U. Città della Salute e della Scienza di Torino, Torino, Italy; 4 Department of Hematology, A.O. Santissimi Antonio e Biagio e C Arrigo, Alessandria, Italy; 5 Division of Hematology, A.O. Santi Croce e Carle, Cuneo, Italy

**Keywords:** Anti-thymocyte globulin, Hematopoietic stem cell transplantation, Matched unrelated donors, GvHD

## Abstract

Despite the widespread use of rabbit anti-thymocyte globulin (ATG) to prevent acute and chronic graft-versus-host disease (aGVHD, cGVHD) after allogeneic hematopoietic cell transplantation (allo-HCT), convincing evidence about an optimal dose is lacking. We retrospectively evaluated the clinical impact of two different ATG doses (5 vs 6–7.5 mg/kg) in 395 adult patients undergoing HSCT from matched unrelated donors (MUD) at 3 Italian centers. Cumulative incidence of aGVHD and moderate-severe cGVHD did not differ in the 2 groups. We observed a trend toward prolonged overall survival (OS) and disease-free survival (DFS) with lower ATG dose (5-year OS and DFS 56.6% vs. 46.3%, *p*=0.052, and 46.8% vs. 38.6%, *p*=0.051, respectively) and no differences in relapse incidence and non-relapse mortality. However, a significantly increased infection-related mortality (IRM) was observed in patients who received a higher ATG dose (16.7% vs. 8.8% in the lower ATG group, *p*=0.019). Besides, graft and relapse-free survival (GRFS) was superior in the lower ATG group (5-year GRFS 43.1% vs. 32.4%, *p*=0.014). The negative impact of higher ATG dose on IRM and GRFS was confirmed by multivariate analysis. Our results suggest that ATG doses higher than 5 mg/kg are not required for MUD allo-HCT and seem associated with worse outcomes.

**Supplementary Information:**

The online version contains supplementary material available at 10.1007/s00277-021-04521-z.

## Introduction

Allogeneic hematopoietic cell transplantation (allo-HCT) is potentially curative for many hematologic malignancies, and human leukocyte antigen (HLA)-matched unrelated donors (MUD) are valid alternatives for patients lacking a HLA-matched related donor (MRD). However, despite improvements in HLA matching techniques that allow a better donor selection, the use of unrelated donors remains associated with an increased risk of graft-versus-host disease (GVHD) [1, 2]. Over the past decades, GVHD prophylaxis strategies have significantly improved, due to the introduction of effective combinations of immunosuppressive agents [3–6]. These regimens have been relatively successful for acute GVHD prophylaxis (aGVHD) but failed to satisfactorily prevent chronic GVHD (cGVHD) [7]. Several prospective randomized trials and subsequent meta-analyses supported the efficacy of prophylactic in vivo T-cell depletion with rabbit anti-thymocyte globulin (ATG) to prevent both aGVHD and cGVHD [8–14]. However, the benefits in terms of prevention of GVHD did not translate into a survival improvement [15–17]. The use of ATG has been associated with delayed immune reconstitution, which might increase the risk of opportunistic infections and impair graft-versus-tumor responses [18].

Two rabbit ATG formulations are used in clinical practice, distinct for antibody quantity and specificity depending on immunization source [19], Jurkat T-cell line (ATLG; Grafalon, previously termed ATG Fresenius, Neovii Biotech, Lexington, MA) or human thymocytes (Thymoglobulin; Genzyme-Sanofi, Cambridge, MA). ATG dosing and formulation significantly differed across studies, and despite its widespread use, convincing evidence about the most appropriate dose is lacking [20]. The identification of the optimal dose of ATG is essential for a conscious use in the context of a tricky balance between prevention of GVHD and immune reconstitution.

While earlier studies employed relatively doses as high as 15mg/kg (Thymoglobulin) [8], more recent reports suggested that lower doses might be more appropriate. Therefore, we performed a retrospective study to assess the impact of ATG doses in patients undergoing allo-HCT from MUD.

## Methods

The current study was performed retrospectively in three Italian Transplant Centers. We included all consecutive adult patients who received allo-HCT for hematological malignancies from 8/8 or 7/8 HLA MUD between January 2005 and December 2016 treated with the ATG brand Thymoglobulin as part of the GVHD prophylaxis regimen.

Clinical data were extracted from electronic database and through electronic and paper charts. Data collected included recipient age, year of transplant and time from diagnosis to transplant, disease status at transplant, conditioning regimen, graft source (bone marrow (BM) or peripheral blood (PB)), donor and recipient sex, HLA matching, GVHD prophylaxis, and ATG dose.

Conditioning regimens were myeloablative (MAC) or reduced intensity (RIC) and defined according to published criteria [21]. The degree of HLA matching was evaluated considering HLA-A, HLA-B, HLA-C, and HLA-DRB1 typing by high-resolution molecular methods. Disease status at transplant was stratified as early or advanced as previously described [22–26].

The European Society for Blood and Marrow Transplantation (EBMT) score was retrospectively calculated according to published criteria [27].

The cut-off date for this analysis was October 2019.

### GVHD prophylaxis

GVHD prophylaxis included cyclosporine (CSA) and short-course methotrexate (MTX, given intravenously on days 1, 3, 6, and 11) or CSA combined with mycophenolate mofetil (MMF).

MTX day 11 dose could be omitted in case of toxicity.

ATG was administered at a total dose ranging from 5 to 7.5 mg/kg body weight; the dose was chosen per physician’s preference. ATG was administered at a dose of 2.5 mg/kg on day −3 and −2 or 0.5mg/kg on day −3, 2 mg/kg on day −2, and 2.5 mg/kg on day −1 (5 mg/kg total dose); 2 mg/kg from day −3 to −1 (6 mg/kg total dose); 3.5 mg/kg on day −3 and −2 (7 mg/kg total dose); and 3.75 mg/kg on day −3 and −2 (7.5 mg/kg total dose).

### Supportive care

Antifungal agents, mostly fluconazole, were routinely administered to all patients. During the neutropenic phase, patients received prophylactic quinolones or cephalosporins. Long-term cotrimoxazole prophylaxis against *Pneumocystis jirovecii* and antiviral prophylaxis with acyclovir were performed in all patients. Cytomegalovirus (CMV) reactivation was routinely monitored at least weekly by polymerase chain reaction (PCR) and/or antigenemia assay. CMV reactivation was pre-emptively treated with ganciclovir or valganciclovir after detection of 2 consecutive positive PCR assay results or 1 positive antigenemia assay in peripheral blood. All patients were weekly monitored for Epstein-Barr virus (EBV) reactivation by PCR in peripheral blood samples. In case of confirmed EBV reactivation, reduction of immunosuppression was performed if GVHD did not concurrently occur and the anti-CD20 monoclonal antibody rituximab was administered. All blood products were irradiated and leukocyte depleted.

### Study endpoints

We aimed to assess whether lower ATG doses (i.e., 5 mg/kg) were equally effective as higher ones in MUD allo-HCT. Primary endpoints were cumulative incidence of aGVHD (grade II–IV and grade III–IV) and moderate/severe cGVHD. Acute GVHD was scored by Glucksberg criteria [28]. Severity of cGVHD was assessed according to the National Institutes of Health (NIH) criteria [29]. Relapse or death from any cause before the occurrence of GVHD were considered as competing events for the incidence of GVHD.

Secondary endpoints included time to neutrophil engraftment, OS, disease-free survival (DFS), refined GVHD/relapse-free survival (GRFS), cumulative incidence of relapse (RI), non-relapse mortality (NRM), infection-related mortality (IRM), and cumulative incidence of CMV and EBV reactivation.

Time to neutrophil engraftment was calculated from transplant day until the first of 3 consecutive days with absolute neutrophil count >500/ul. OS was defined as the time from allo-HCT to death, regardless of the cause while DFS as the time from allo-HCT to relapse or death from any cause, whichever occurred first. Refined GRFS was defined as the absence of grade III–IV aGVHD, severe cGVHD, relapse, and death as defined by Ruggeri et al. [30] RI was defined as time from allo-HCT to relapse. NRM was defined as death in remission. NRM was regarded as a competing event for relapse and relapse as a competing event for NRM. IRM was defined as death with infection as the primary cause of death, considering as competing events for IRM relapse, GVHD, and other causes of death [31].

The cumulative incidence of CMV infection was estimated as an event of interest and death without CMV infection as a competing event. Death without EBV reactivation was a competing event for the cumulative incidence of EBV. Time to infection was calculated from transplant day until the day of first positive result. Follow-up for survival was censored when the patient was last verified to be alive. Patients who received more than one transplant were censored as alive at day of the subsequent transplant.

### Statistical analysis

Variables are reported as medians and interquartile ranges (IQR) for continuous variables and as numbers and proportions for categorical ones, respectively. Fisher’s exact and Mann-Whitney *U* test were used to compare categorical and continuous variables, respectively.

Follow-up duration was calculated with the inverse method [32].

OS, DFS, and GRFS were estimated using the Kaplan-Meier method and compared using the log-rank test. Cumulative incidence functions were estimated using appropriate competing risks analyses and compared by Gray’s test.

Multivariate analyses were performed using Cox proportional hazards models, followed by backward stepwise selection. The following clinical variables were tested as risk factors: age at allo-HCT, sex, disease status (active disease vs. complete remission), intensity of conditioning regimen (MAC vs. RIC), graft source (PB vs. BM), donor and recipient sex mismatch (female to male vs. other), HLA match (8/8 vs. 7/8), and ATG dose (higher dose vs. lower dose).

The proportional hazard (PH) assumption was validated by visual inspection and testing of Schöenfeld residuals [33]. Variance inflation factor (VIF) was used to rule out multicollinearity considering VIF > 4 as unacceptable [34].

All reported *p* values were two-sided at the conventional 0.05 significance level. Confidence intervals were reported at a 95% level. Statistical analyses were performed using STATA 12.1 (Stata Corporation, College Station, Texas) and NCSS 11 Statistical Software (NCSS, LCC. Kaysville, UT, USA)

## Results

### Patient characteristics

Three hundred ninety-five patients (males 54.7%) with a median age at allo-HCT of 51.4 years (IQR 40.8–59.8 years) were included. Baseline patient characteristics are summarized in Table 1.

**Table 1:** Baseline patient, disease, and transplant characteristics

	All	Lower dose group	Higher dose group	*p*
Patients	395	197	198	
Age*	51.4 (20.7–69.4)	52.4 (20.7–69.4)	50.4 (20.7–66.8)	**0.043**
Male sex	216 (54.7%)	99 (50.3%)	117 (59%)	0.09
Hematological disease				0.13
ALL-B/ALL-T	52 (13.2%)	23 (11.7%)	29 (14.7%)	
AML/MDS	199 (50.4%)	111 (56.3%)	88 (44.4%)	
MPN	33 (8.3%)	14 (7.1%)	19 (9.6%)	
LPD	111 (28.1%)	49 (24.9%)	62 (31.3%)	
Disease status at allo-HCT				0.11
CR	265 (67.1%)	140 (71.1%)	125 (63.1%)	
Active disease	130 (32.9%)	57 (28.9%)	73 (36.9%)	
EBMT score				0.54
Low risk	154 (39%)	82 (41.8%)	72 (36.4%)	
Intermediate	93 (23.5%)	44 (22%)	49 (24.7%)	
High	148 (37.5%)	71 (36.2%)	77 (38.9%)	
Conditioning regimen				**<.001**
MAC	258 (65.3%)	154 (78.2%)	107 (54%)	
RIC	137 (34.7%)	43 (21.8%)	91 (46%)	
Stem cell source				0.56
BM	55 (13.9%)	25 (12.7%)	30 (15.15%)	
PB	340 (86.1%)	172 (87.3%)	168 (84.85%)	
GVHD prophylaxis				0.13
CSA+ MTX	372 (94.2%)	190 (96.5%)	182 (91.9%)	
CSA+ MMF	23 (5.8%)	7 (3.5%)	16 (8.1%)	
Female to male D/R	53 (13.4%)	25 (12.7%)	28 (14.1%)	0.77
HLA disparity				**<.001**
No mismatch	255 (64.6%)	147 (74.6%)	108 (54.5%)	
1 HLA locus mismatch	140 (35.4%)	50 (25.4%)	90 (45.5%)	

Values in bold are significant *p* values

*Median (range); otherwise, data are presented as number (%). *ALL* acute lymphoid leukemia, *AML* acute myeloid leukemia, *CR* complete remission, *D/R* donor/recipient, *LPD* lymphoproliferative disorders, *MAC* myeloablative conditioning, *MDS* myelodysplastic syndrome, *MPN* myeloproliferative neoplasms, *RIC* reduced intensity conditioning, *BM* bone marrow, *PB* peripheral blood stem cells, *CSA* ciclosporin, *MTX* methotrexate, *MMF* mycophenolate mofetil

One-hundred and ninety-seven patients (49.9%) received ATG at total dose of 5 mg/kg (lower dose group) and 198 (50.1%) a higher dose, ranging from 6 to 7.5 mg/kg (higher dose group) (Supplementary Table 1). Patient’s characteristics, including graft source (PB in over 80% of the cases), disease status at allo-HCT, and GVHD prophylaxis (mainly CSA and short MTX course), were equally distributed in both groups, although MTX day 11 dose was omitted more often in the higher ATG dose group (54.1 vs 30.4%, *p*<0.001). Myeloablative conditioning regimens were used more frequently in the lower dose group (78.2% vs. 54.0%, *p*<0.001), and a higher proportion of HLA mismatched transplants was found in the higher dose one (45.5% vs. 25.4%, *p*<0.001). In addition, there was a slight imbalance of age (median age at transplant 52.4 years vs. 50.4 years in the lower and higher ATG dose group, respectively, *p*=0.043).

Median follow-up was 81.5 months (IQR 50.2–119.3 months).

### Engraftment and GVHD

Median time to neutrophil engraftment was 17 days (IQR 14–20 days), without difference according to ATG dose (*p*=0.85). Only one patient in the low-dose group and 3 patients in the high dose one failed to engraft.

**Fig. 1:**
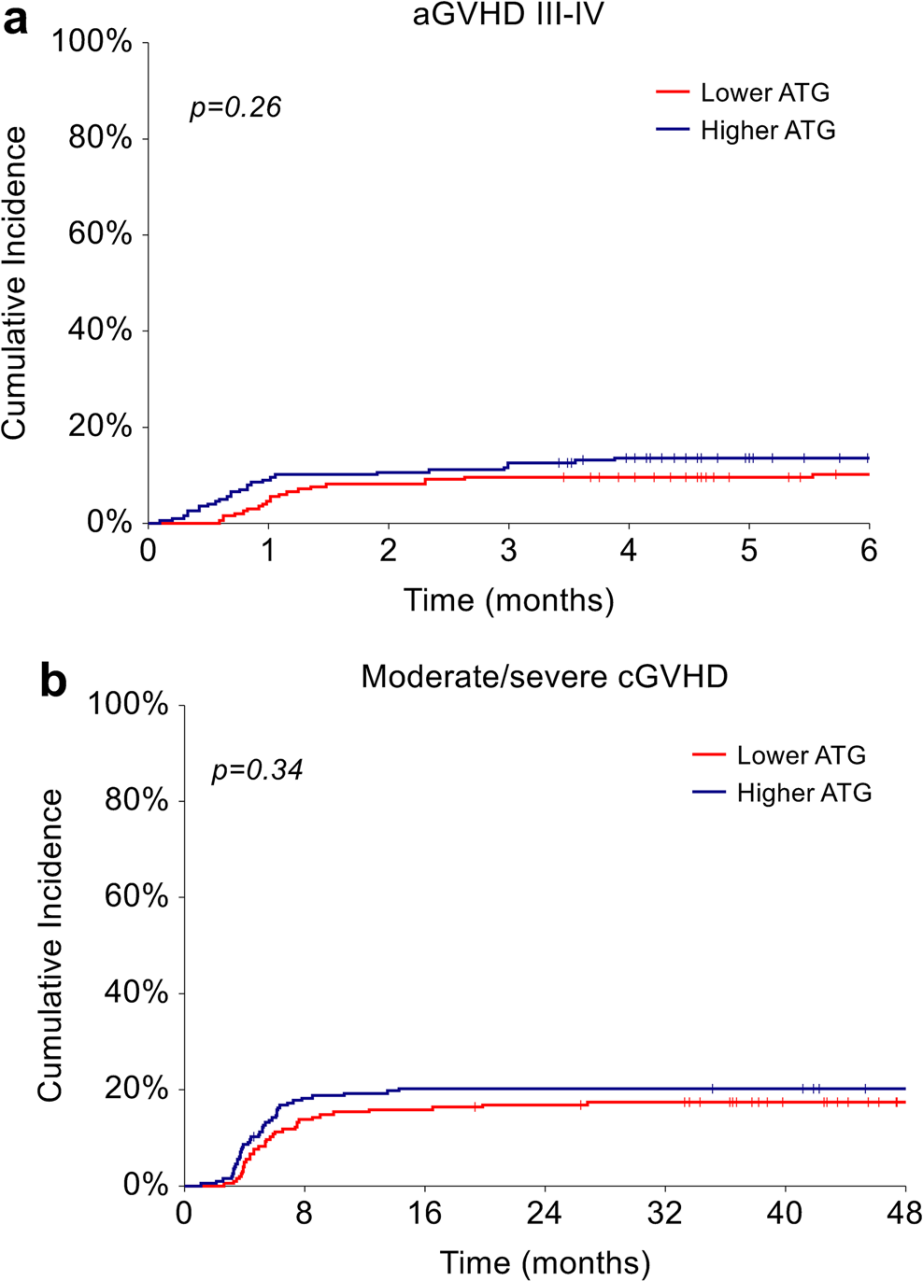
Cumulative incidence of GVHD. (**a**) Cumulative incidence of aGVHD grades III to IV and (**b**) cumulative incidence of moderate to severe cGVHD in patients treated with lower dose (5 mg/kg) and higher dose (6–7.5 mg/kg) of ATG

Day 180 cumulative incidences of grade II–IV aGVHD and grade III–IV aGvHD were similar between the groups, 28.6% vs. 33.9% (*p*=0.18) and 10.2% vs. 13.7% (*p*=0.26), in the lower and higher dose groups, respectively (Fig. 1a). Likewise, 4-year moderate-severe cGVHD did not differ between ATG doses (17.4% in the lower dose group vs. 20.3% in the higher dose group, *p*=0.34) (Fig. 1b and Supplementary Table 2).

By multivariate analysis, the presence of a HLA-mismatch was the only factor with a significant impact on the cumulative incidence of II–IV aGVHD (sub-hazard ratio [sHR]= 1.64, 95% CI 1.15–2.33, *p*=0.006) and III–IV aGVHD (sHR=3.17, 95% CI 1.76–5.70, *p*<0.001), regardless of ATG dose. Conversely, no independent predictive factor for moderate/severe cGVHD was identified, with female donors into male recipients reaching borderline significance (*p*=0.052). Of note, mismatched donors did not significantly influence the risk of cGVHD (*p*=0.35) (Table 2 and Supplementary Table 4).

**Table 2:** Multivariate analysis of outcomes based on patient, disease, and transplant characteristics

Variable	Grade III–IV aGVHD				cGVHD†*	OS				GRFS				IRM			
	Univariate	Multivariate			Univariate	Univariate	Multivariate			Univariate	Multivariate			Univariate	Multivariate		
	*p*	sHR	95% CI	*p*	*p*	*p*	HR	95% CI	*p*	*p*	HR	95% CI	*p*	*p*	sHR	95%CI	*p*
Age, years§	0.23				0.76	0.12				0.55				**0.004**	1.04	1.02–1.07	**0.002**
Male gender	0.82				0.21	0.70				0.72				0.26			
Active disease	0.12				0.07	**<0.001**	2.13	1.61–2.82	**<0.001**	**<0.001**	2.11	1.61–2.76	**<0.001**	**<0.001**	2.46	1.41–4.28	**0.002**
RIC	0.78				0.13	0.27				0.99	0.72	0.54–0.96	**0.023**	**0.005**			
BM graft	0.80				0.83	0.28				0.17				0.22			
Female to male D/R	0.52				0.052	0.61				0.77				0.73			
HLA mismatch	**<0.001**	3.17	1.76–5.70	**<0.001**	0.35	0.12				0.073				0.29			
High ATG dose	0.25				0.35	0.052				**0.014**	1.37	1.06-1.78	**0.015**	**0.019**	2.05	1.14–3.68	**0.016**

Values in bold are significant *p* values

*Moderate–severe

†No significant association by multivariate analysis

§As continuous variable, analyzed by Cox model; *BM* bone marrow, *D/R* donor/recipient, *GVHD* Graft-versus-host disease, *OS* overall survival, *GRFS* GVHD/relapse-free survival, *HR* hazard ratio, *IRM* infection-related mortality, *RIC* reduced intensity conditioning, *sHR* sub-hazard ratio

### Survival outcomes

**Fig. 2:**
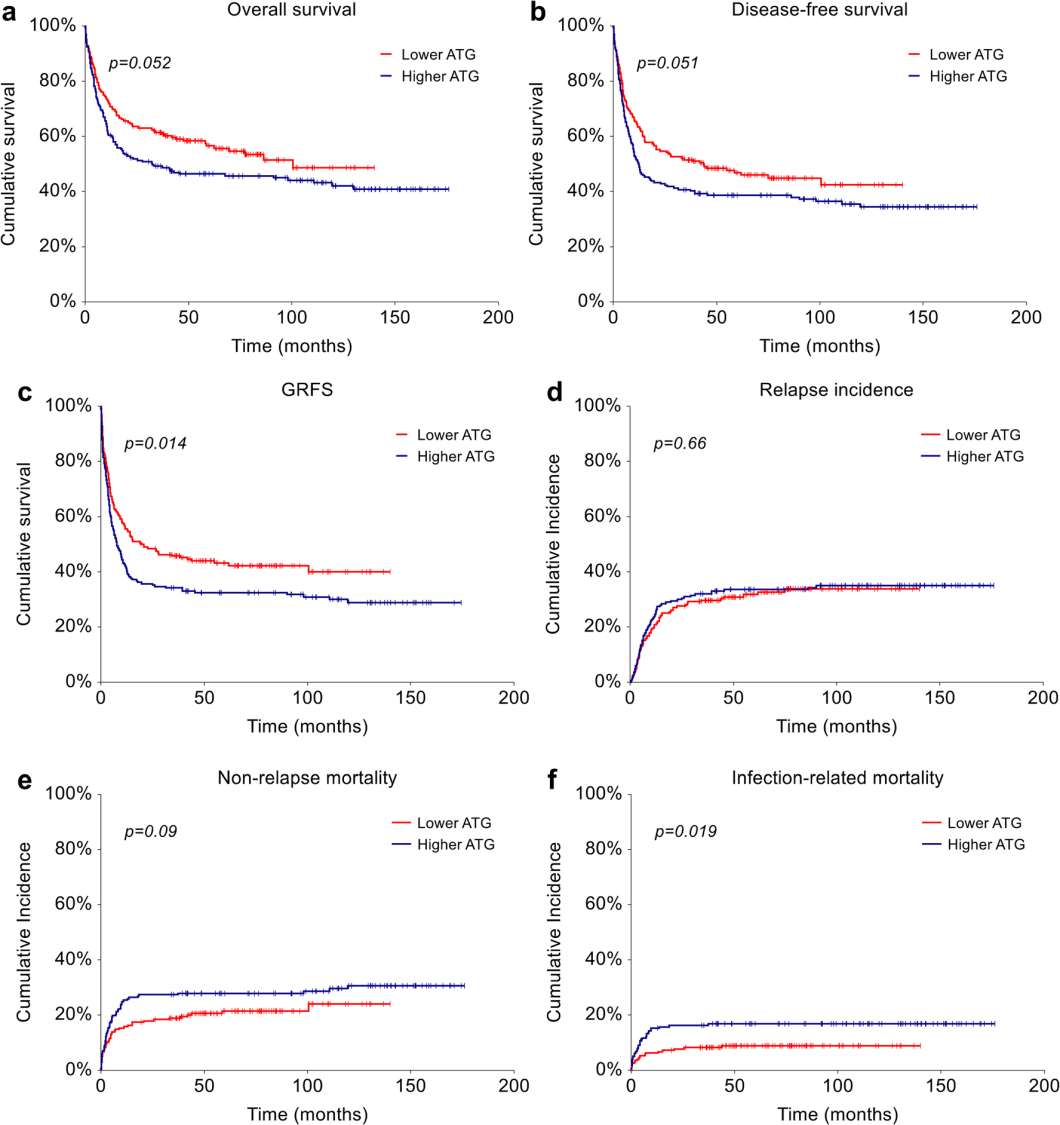
Impact of ATG dose on transplant outcomes. (**a**) OS, (**b**) DFS, (**c**) GRFS, (**d**) RI, (**e**) NRM, and (**f**) IRM in patients treated with lower dose (5 mg/kg) and higher dose (6–7.5 mg/kg) of ATG

Estimated median OS and DFS for the entire cohort were 77.3 months and 13.0 months, respectively, with a trend toward an advantage for low ATG dose compared to the higher one (5-year OS 56.6% vs. 46.3%, *p*=0.052, and 5-year DFS 46.8% vs. 38.6%, *p*=0.051, respectively) (Fig. 2a–b). Both by univariate and by multivariate analysis, the only factor significantly associated with OS and DFS was disease status at allo-HCT (HR=2.13, 95% CI 1.61–2.82, *p*<0.001 and HR=2.38, 95% CI 1.83–3.08, *p*<0.001, respectively).

GRFS was significantly improved in the lower ATG dose group (5-year GRFS 43.1% vs. 32.4% in the higher dose group, *p*=0.014) (Fig. 2c). By multivariate analysis, higher dose of ATG remained significantly associated with a reduced GRFS (HR=1.37, 95% CI 1.06–1.78, *p*=0.015), along with active disease at transplant (HR=2.11, 95% CI 1.61–2.76, *p*<0.001). Conversely the use of RIC was associated with an improved GRFS (HR=0.72, 95% CI 0.54–0.96, *p*=0.023) (Table 2 and Supplementary Table 4).

### Relapse incidence and non-relapse mortality

Relapse risk did not differ according to ATG dose (5-year RI 31.7% and 33.6% in the lower and higher ATG dose group, respectively, *p*=0.66) (Fig. 2d). By multivariate analysis, only age and active disease had an impact on RI (sHR=0.98, 95% CI 0.97–0.99, *p*<0.001, and sHR=2.06, 95% CI 1.46–2.92, *p*<0.001, respectively).

Disease relapse was the most common cause of death in both groups, while the main causes of NRM were infections (*N*=18, 46.1% vs. *N*=34, 60.7%, *p*=0.018), followed by organ failure (*N*= 12, 30.8% vs. *N*=13, 23.2%, *p*=ns) and GVHD (*N*=9, 23.1% vs. *N*=7, 12.5%, *p*=ns) in the lower and higher ATG dose groups, respectively. Two patients who received higher ATG doses died from road accidents. Higher ATG dose was associated with a non-significant trend toward an increased NRM (5-year NRM 27.9% vs. 21.5%, *p*=0.094, Fig. 2e), which was significantly influenced by age and active disease at transplant (sHR=1.04, 95% CI 1.02–1.06, *p*<0.001, and sHR=1.69, 95% CI 1.14–2.50, *p*=0.01, respectively) by multivariate analysis (Supplementary Table 4).

### Infection-related mortality and viral infections

A statistically significantly higher probability of IRM was observed in patients who received a higher ATG dose (16.7% vs. 8.8%, sHR=2.01, 95% CI 1.12–3.61, *p*=0.019, Fig. 2f). The adverse impact of higher ATG dose on IRM was confirmed by multivariate analysis (sHR=2.05, 95% CI 1.14–3.68, *p*=0.016), along with higher age (sHR=1.04, 95% CI 1.02–1.07, *p*=0.002) and active disease at transplantation (sHR=2.46, 95% CI 1.41–4.28, *p*=0.002) (Table 2).

Detailed data on CMV and EBV reactivations was missing in 47 patients. Day 100 cumulative incidence of CMV reactivation was 32.7% in lower ATG dose group and 35.6% in the higher dose group (*p*=0.30). Median day of CMV reactivation was day 39 (IQR 29–48 days) in the lower dose group and day 30 in the higher dose group (IQR 21–43 days), without difference according to ATG dose (*p*=0.22). EBV reactivation occurred in 10.7% patients in the lower ATG dose group and 11.1% in the higher dose one (*p*=0.95). Pneumonia accounted for 15 (28.8%) infection-related deaths, probable/proven invasive fungal infection for 17 (32.7%), septic shock for 10 (19.2%), central nervous system infection for 4 (7.7%), and other/unknown infection for 6 (11.6%). Causes of IRM according to ATG dose are summarized in Supplementary Table 5.

### Subgroup analyses

Given the major impact of disease status at allo-HCT on clinical outcomes, we conducted a subgroup analysis on patients in remission at the time of transplant (*n*=265). ATG dose did not impact on grade III–IV aGVHD (*p*=0.34) and moderate-severe cGVHD (*p*=0.67). Median OS was not reached in these patients and was negatively affected by higher ATG dose (5-year OS 53.0% vs. 68.6%, HR=1.58, 95% CI 1.08–2.32, *p*=0.018). Similarly, DFS was inferior with higher ATG dose (HR=1.47, 95% CI 1.04–2.1, *p*=0.03), as well as GRFS (HR=1.51, 95% CI 1.09–2.1, *p*=0.013).

Next, we considered patients who received a RIC (*n*=134), which was associated with a significantly higher probability of GRFS in our cohort. Higher ATG dose was detrimental in this subgroup of patients in terms of GRFS (5-year GRFS 30.7% vs. 50.6%, HR=1.85, 95% CI 1.13–3.04, *p*=0.013). Median OS of patients who received a RIC was 45.7 months, without difference according to ATG dose (*p*=0.11).

Finally, we explored the role of ATG dose in HLA-matched patients (*n*=255), but we did not find a significant impact on any outcome, while in the subgroup of HLA-mismatched transplants (*n*=140), a trend toward a better DFS, GRFS, and IRM was observed in patients receiving a lower ATG dose.

**Fig. 3:**
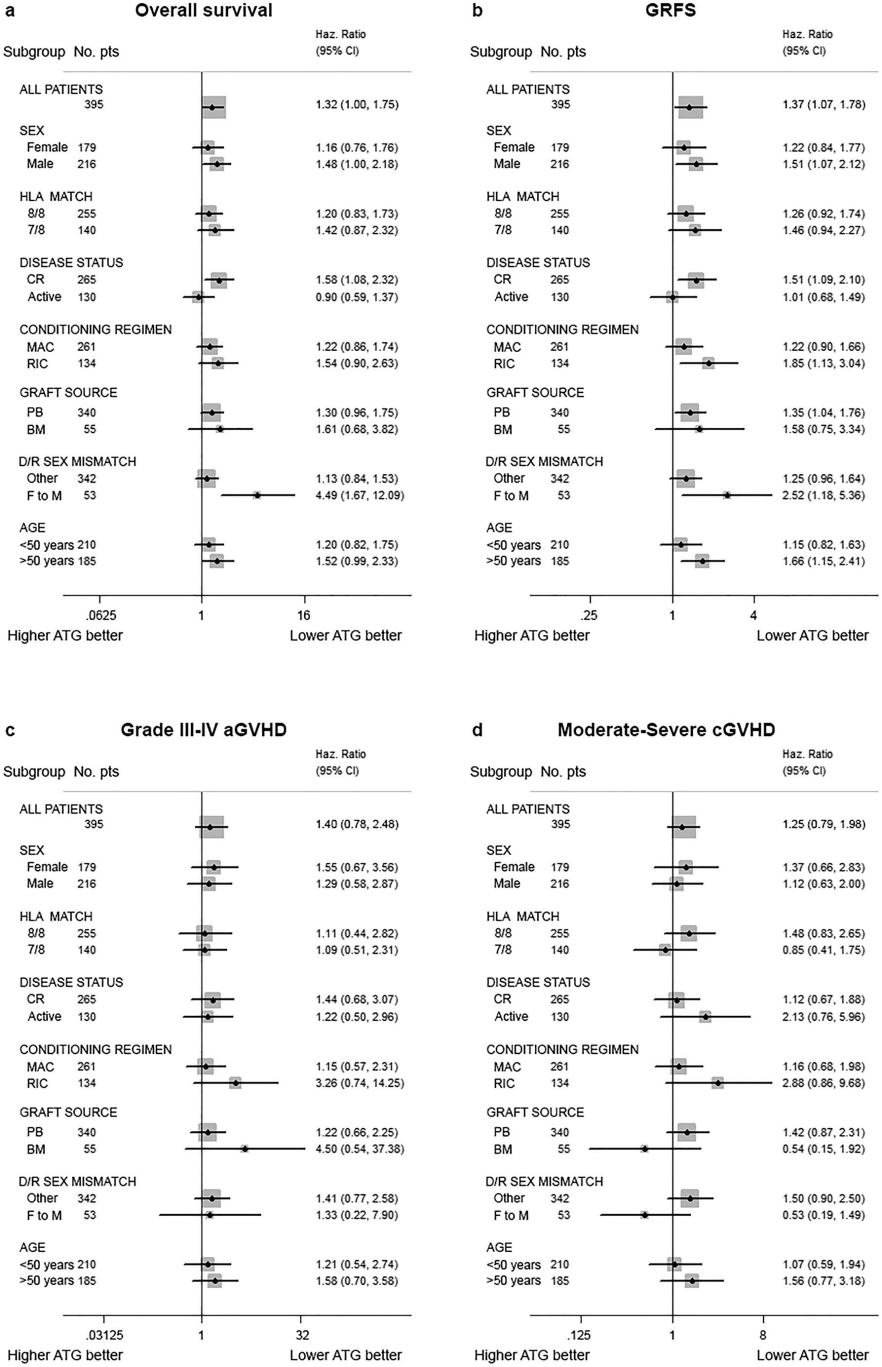
Subgroup analyses. Impact of ATG dose on (**a**) OS, (**b**) GRFS, (**c**) grade III–IV aGVHD, and (**d**) moderate/severe cGVHD according to baseline patients’ characteristics

The impact of ATG dose on different clinical outcomes in the most relevant subgroups is summarized in Fig. 3 and Supplementary Figure 1 to 3.

## Discussion

ATG showed to be protective against GVHD, and it is recommended as part of GVHD prophylaxis both in MUD and MRD transplants [35]. However, the determination of the optimal dose may be difficult, given the complex interplay of factors that have an impact on the risk of complications after allo-HCT. Indeed, when selecting the dose of ATG, both transplant-related factors including graft source, donor and conditioning regimen intensity, and disease-related factors should be balanced.

Recently, a consensus-based recommendation by an international expert panel suggested the use of 30 mg/kg and 60 mg/kg of ATLG for sibling and unrelated MAC transplants, respectively, or 4.5–7.5 mg/kg of ATG [36].

In the randomized trial conducted by Finke et al., the addition of ATLG was associated with reduced extensive cGVHD, improved GRFS, and survival free of immunosuppressive therapy compared to standard prophylaxis, with no effect on relapse mortality and survival [37]. Of note, similar results were found by Kroger et al. in patients receiving MRD transplants with PB as graft source. The probability of CSA discontinuation at 1 year was 39% in the standard group and 91% in the ALTG group [11]. Even though the data observed for the available brands of ATG are difficult to compare because of their different immunologic and pharmacokinetics (PK) properties [38], the addition of ATG as GVHD prophylaxis showed similar results in cGVHD prevention and improvement of GRFS [35, 36].

Indeed, our study was limited by its retrospective nature and by the heterogeneous populations in terms of disease characteristics and transplantation modalities. However, we included a relatively large patient number with a rather long follow-up, which allowed a reliable estimation of ATG effects on long-term clinical outcomes at our centers. The two ATG dose groups showed some imbalances that were, however, adjusted by multivariate and subgroup analyses. MTX day 11 dose was omitted more often in the higher ATG dose group, but it did not exert an impact on any of the clinical outcome analyzed. Furthermore, the heterogeneity of high dose group did not translate into significant differences on key clinical outcomes in patients receiving 6, 7, or 7.5 mg/kg of ATG (data not shown).

Overall, ATG dose did not appear to impact on aGVHD (any grade) and moderate-severe cGVHD. With the limitation of different ATG brands, these results are similar to those reported in the randomized trial by Walker et al. and in the extended follow-up by Finke et al. with ATLG [37, 39, 40].

Despite the use of ATG, HLA mismatch remained the only independent risk factor for the development of grade II–IV and severe aGVHD, which was not overcome by higher ATG doses. By contrast, both doses of ATG overcame the negative impact of HLA disparity on cGVHD. The complex immunological properties of ATG could explain the reduction of cGVHD risk. As a matter of fact, in addition to extensive T-cell depletion, ATG affects different immune effectors involved in cGVHD development, such as B lymphocytes and dendritic cells. Moreover, ATG was shown to induce regulatory T lymphocyte expansion [41, 42].

Data from the randomized study by Soiffer et al. hinted at a decreased progression-free survival and OS in patients receiving in vivo T-cell depletion [43], but this finding was not confirmed by other randomized studies [8–12]. In our analysis, higher ATG dose appeared to impair DFS and OS, particularly in the subgroup of patients in complete remission at allo-HCT.

Infectious complications remain a major concern associated with T-cell depletion; in particular, higher ATG dose (15 mg/kg) has been associated with an increased risk of lethal infections [8]. Similarly, in our cohort, higher ATG dose conferred an increased risk of infectious death, independently from other known prognostic factors.

In our study, although GVHD incidence did not significantly differ in the two groups, lower ATG dose was associated with a significantly improved GRFS, which was confirmed by multivariate analysis. This finding is noteworthy, because GRFS identifies patients who survive without relapse or severe complications and who may enjoy a high quality of life. The improvement on the composite outcome of GRFS in the lower ATG dose group was probably driven by the reduction of IRM, but a meaningful albeit not statistically significant reduction of GVHD ad relapses could have contributed to explaining this result.

The impact of ATG on clinical outcomes might also be affected by the intensity of conditioning regimen [44, 45]. The role of ATG in RIC regimens remains to be determined, as only one randomized trial included RIC transplants and conflicting results were reported in retrospective studies [36, 39]. However, it was recommended that ATG should be considered in this setting as well, although a higher risk of relapse should be taken into account [36]. In our study, the use of RIC regimens correlated with improved GRFS, and the impact of ATG doses on clinical outcomes were consistent with those observed in the entire population. Feasibility and efficacy are in line with results observed in previous retrospective comparative studies [46].

Our results obtained with Thymoglobulin cannot be easily applied and generalized to different brands of ATG; nevertheless, nonrandomized comparison between standard dose and a lower dose of ATLG showed similar outcomes [47]. To improve the use of ATG in the future, a standard dose per body weight could be replaced by an individualized approach to ATG dosing based on PK and pharmacodynamic (PD) models. Admiraal et al. showed that PK of ATG was dependent on recipient’s absolute lymphocyte count at time of infusion and cellular-target dosing provided an optimal ATG area under curve that resulted in lower incidence of GvHD and graft failure, while higher exposure led to worse immune reconstitution [48]. Prospective randomized trials are essential to develop and validate PK-based models [49, 50].

Recently, a matched-pair analysis of the Acute Leukemia Working Party of the EBMT compared the use of post-transplant cyclophosphamide (PTCy) versus ATG in the setting of 9/10 MMUD allo-HCT. A significantly lower incidence of severe aGVHD was observed with PTCy. The use of the latter was also associated with superior survival outcomes in terms of leukemia-free survival and GRFS [51]. A prospective GITMO study (NCT03270748) is currently investigating the efficacy of PTCy in 9/10 MMUD transplants for acute myeloid leukemia and myelodysplastic syndrome, and results are eagerly awaited.

In conclusion, in our study, an ATG dose of 5 mg/kg was as effective as higher doses for GVHD prevention after MUD allo-HCT. A higher dose did not confer any additional benefit; conversely it appeared to be associated with increased IRM and reduced GRFS. ATG doses and preferable T-cell depletion approaches should be addressed in prospective randomized studies.

## Supplementary information

ESM 1 (DOC 772 kb)

## Data Availability

Data is available upon reasonable request to the corresponding author.
