# Supplementary material for: Impact of anti-thymocyte globulin dose for graft-versus-host disease prophylaxis in allogeneic hematopoietic cell transplantation from matched unrelated donors: a multicenter experience
Source: Ann Hematol. 2021 May 4;100(7):1837–47. doi: 10.1007/s00277-021-04521-z (PMC8195753; doi:10.1007/s00277-021-04521-z)
Supplement: Supplementary file 1 — (DOC 772 kb) [file 277_2021_4521_MOESM1_ESM.doc]

**Impact of Anti-thymocyte Globulin Dose for Graft-versus-host Disease Prophylaxis in Allogeneic Hematopoietic Cell Transplantation from Matched Unrelated Donors: A Multicenter Experience.**

Sara Butera*^1,2^, Marco Cerrano*^3^, Lucia Brunello^1,4^, Chiara Maria Dellacasa^1^, Danilo Giuseppe Faraci^1,2^, Sara Vassallo^1,2^, Nicola Mordini^5^, Roberto Sorasio^5^, Francesco Zallio^4^, Alessandro Busca^1^, Benedetto Bruno^1,2^ and Luisa Giaccone^1,2^.

**Supplementary Appendix**

**Supplementary Tables 1-5: pages 2-4**

**Supplementary Figure 1-3: pages 5-7**

**Supplementary Table 1. ATG dose of the study population.**

| ATG dose, mg/kg | Number of patients | % |
| --- | --- | --- |
| 5 | 197 | 49.9 |
| 6 | 19 | 4.8 |
| 7 | 132 | 33.4 |
| 7.5 | 47 | 11.9 |

Supplementary Table 2. Cumulative incidence of GVHD according to ATG dose.

| *GVHD (95%CI)* | *Lower dose* | *Higher dose* | *p value* |
| --- | --- | --- | --- |
| 180d aGVHD(II-IV) | 28.6 (22.9-35.7) | 33.9 (27.9-41.1) | 0.18 |
| 180d aGVHD(III-IV) | 10.2 (6.7-15.5) | 13.7 (9.6-19.4) | 0.26 |
| 4y-cGVHD moderate/severe | 17.4 (12.8-23.6) | 20.3 (15.3-26.7) | 0.34 |

**Supplementary Table 3. Survival Outco**mes.

| *5-year Outcomes (95% CI)* | *Lower dose* | *Higher dose* | *p value* |
| --- | --- | --- | --- |
| OS | 56.6 (49.4-63.8) | 46.3 (39.3-53.3) | 0.052 |
| RFS | 46.8 (39.6-54.0) | 38.6 (31.7-45,4) | 0.051 |
| GRFS | 43.1 (36-50.2) | 32.4 (25.9-39) | **0.014** |
| RI | 31.7 (25.7-39) | 33.6 (27.6-40.9) | 0.66 |
| NRM | 21.5 (16.3-28.3) | 27.9 (22.3-34.9) | 0.09 |
| IRM | 8.8 (5.5-13.9) | 16.7 (12.3-22.8) | **0.019** |

**Supplementary Table 4. Multivariate analysis of outcomes based on patient, disease, and transplant characteristics.**

| **Variable** | **Grade II-IV aGVHD** | | | | **DFS** | | | | | **RI** | | | | **NRM** | | | |  |
| --- | --- | --- | --- | --- | --- | --- | --- | --- | --- | --- | --- | --- | --- | --- | --- | --- | --- | --- |
|  | *Univariate* | *Multivariate* | | | *Univariate* | *Multivariate* | | | | *Univariate* | *Multivariate* | | | *Univariate* | *Multivariate* | | |  |
|  | *p* | *sHR* | *95%CI* | *p* | *p* | *HR* | *95%CI* | | *p* | *p* | *sHR* | *95%CI* | *p* | *p* | *sHR* | *95%CI* | *p* |  |
| **Age, years§** | 0.15 |  |  |  | 0.86 |  |  |  | | **<0.001** | 0.98 | 0.97-0.99 | **<0.001** | **<0.001** | 1.04 | 1.02-1.06 | **<0.001** |  |
| **Male Gender** | 0.81 |  |  |  | 0.99 |  |  |  | | 0.25 |  |  |  | 0.29 |  |  |  |  |
| **Active Disease** | 0.65 |  |  |  | **<0.001** | 2.38 | 1.83-3.08 | **<0.001** | | **<0.001** | 2.06 | 1.46-2.92 | **<0.001** | **0.01** | 1.69 | 1.14-2.50 | **0.01** |  |
| **RIC** | 0.99 |  |  |  | 0.45 |  |  |  | | 0.56 |  |  |  | 0.14 |  |  |  |  |
| **BM graft** | 0.11 |  |  |  | 0.21 |  |  |  | | 0.95 |  |  |  | 0.20 |  |  |  |  |
| **Female to Male D/R** | 0.49 |  |  |  | 0.85 |  |  |  | | 0.91 |  |  |  | 0.91 |  |  |  |  |
| **HLA Mismatch** | **0.006** | 1.64 | 1.15-2.33 | **0.006** | 0.15 |  |  |  | | 0.86 |  |  |  | 0.17 |  |  |  |  |
| **High ATG Dose** | 0.17 |  |  |  | 0.051 |  |  |  | | 0.66 |  |  |  | 0.094 |  |  |  |  |
| §as continuous variable, analysed by Cox model; BM, bone marrow; D/R, donor/recipient; GVHD, Graft-versus-host disease; OS, Overall Survival; GRFS, GVHD/Relapse-free survival; HR, Hazard Ratio; IRM, Infection-related mortality; RIC, reduced intensity conditioning; sHR, sub-Hazard Ratio | | | | | | | | | | | | | | | | | |  |

**Supplementary Table 5. Causes of IRM according to ATG dose.**

|  |  |  | *Lower dose* | *Higher dose* |
| --- | --- | --- | --- | --- |
| Pneumonia | | 15 (28.8%) | 2 | 13 |
| Probable/proven IFI | | 17 (32.7%) | 8 | 9 |
| Septic shock | | 10 (19.2%) | 6 | 4 |
| Viral CNS infection | | 4 (7.7%) | 0 | 4 |
| Other site/unknown origin | | 6 (11.6%) | 2 | 4 |
| Data are presented as number (%). IFI, invasive fungal infection; CNS, central nervous system. | | | |  |

**Supplementary Figure 1. Subgroup analyses of (a) aGVHD (b) DFS (c) NRM (d) IRM**

| 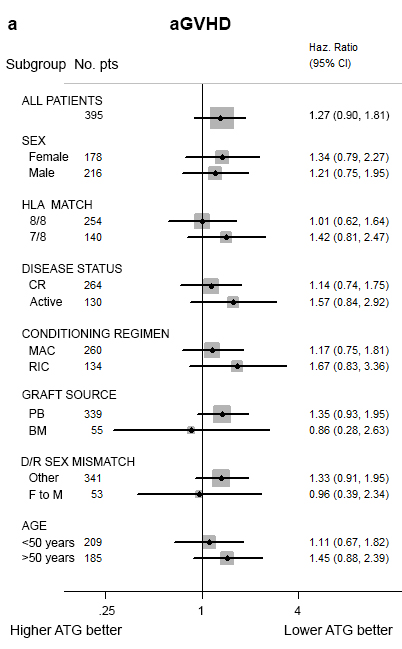 | **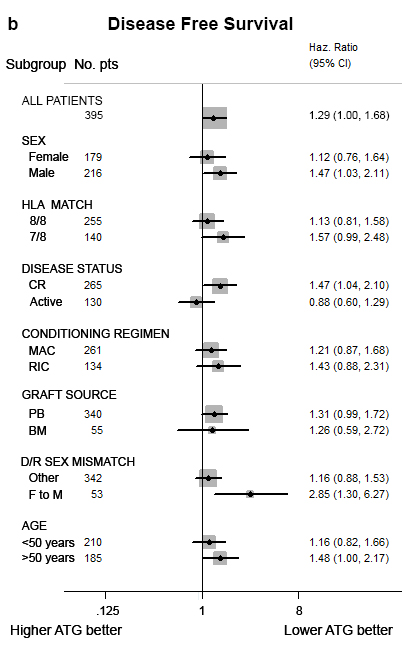** |
| --- | --- |
| **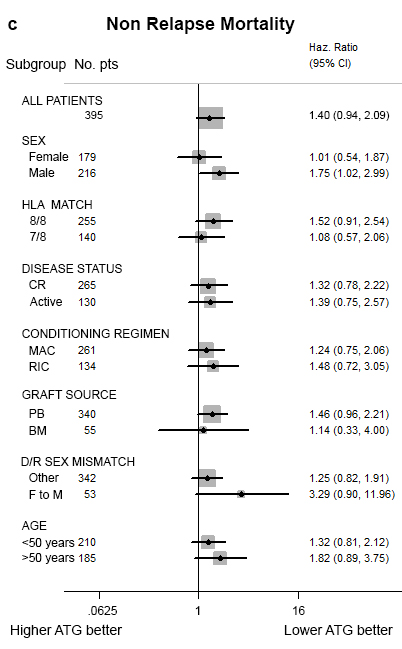** | **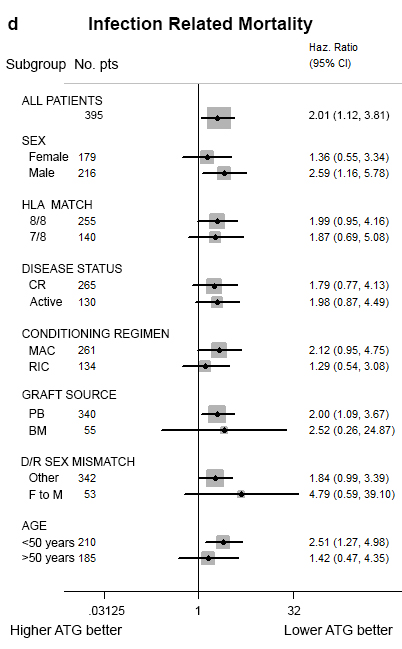** |
| aGVHD, acute graft-versus-host disease; CR, complete remission; MAC, myeloablative conditioning; RIC, reduced intensity conditioning; PB, peripheral blood; BM, bone marrow; D/R, donor/recipient; F to M, female to male | |

**Supplementary Figure 2. Clinical outcomes of HLA identical transplants according to ATG dose (a) cumulative incidence of aGVHD grades II to IV, (b) cumulative incidence of moderate to severe cGVHD, (c) OS (d) DFS (e) GRFS (f) IRM.**

|  |
| --- |

**Supplementary Figure 3. Clinical outcomes of HLA mismatched transplants according to ATG dose (a) cumulative incidence of aGVHD grades II to IV, (b) cumulative incidence of moderate to severe cGVHD, (c) OS (d) DFS (e) GRFS (f) IRM.**

|  |
| --- |
